# Epistatic study reveals two genetic interactions in blood pressure regulation

**DOI:** 10.1186/1471-2350-14-2

**Published:** 2013-01-08

**Authors:** Ndeye Coumba Ndiaye, El Shamieh Said, Maria G Stathopoulou, Gérard Siest, Michael Y Tsai, Sophie Visvikis-Siest

**Affiliations:** 1 “Cardiovascular Genetics” Research Unit, EA-4373, University of Lorraine, 30 rue Lionnois – 54000, Nancy, France; 2 Department of Laboratory Medicine & Pathology, University of Minnesota, Minneapolis, MN, 55455-0392, USA; 3 Department of Internal Medicine and Geriatrics, CHU Nancy-Brabois, France

**Keywords:** Blood pressure, Epistasis, Single nucleotide polymorphism, Epidemiology

## Abstract

**Background:**

Although numerous candidate gene and genome-wide association studies have been performed on blood pressure, a small number of regulating genetic variants having a limited effect have been identified. This phenomenon can partially be explained by possible gene-gene/epistasis interactions that were little investigated so far.

**Methods:**

We performed a pre-planned two-phase investigation: in phase 1, one hundred single nucleotide polymorphisms (SNPs) in 65 candidate genes were genotyped in 1,912 French unrelated adults in order to study their two-locus combined effects on blood pressure (BP) levels. In phase 2, the significant epistatic interactions observed in phase 1 were tested in an independent population gathering 1,755 unrelated European adults.

**Results:**

Among the 9 genetic variants significantly associated with systolic and diastolic BP in phase 1, some may act through altering the corresponding protein levels: SNPs rs5742910 (P_adjusted_≤0.03) and rs6046 (P_adjusted_ =0.044) in *F7* and rs1800469 (P_adjusted_ ≤0.036) in *TGFB1*; whereas some may be functional through altering the corresponding protein structure: rs1800590 (P_adjusted_ =0.028, SE=0.088) in *LPL* and rs2228570 (P_adjusted_ ≤9.48×10^-4^) in *VDR*. The two epistatic interactions found for systolic and diastolic BP in the discovery phase: *VCAM1* (rs1041163) * *APOB* (rs1367117), and *SCGB1A1* (rs3741240) * *LPL* (rs1800590), were tested in the replication population and we observed significant interactions on DBP. *In silico* analyses yielded putative functional properties of the SNPs involved in these epistatic interactions trough the alteration of corresponding protein structures.

**Conclusions:**

These findings support the hypothesis that different pathways and then different genes may act synergistically in order to modify BP. This could highlight novel pathophysiologic mechanisms underlying hypertension.

## Background

Blood pressure (BP) is a continuous, consistent and modifiable risk factor for cardiovascular diseases (CVD) such as ischemic heart disease (IHD) and stroke which are major causes of mortality and morbidity worldwide [1]: population-based studies showed that 972 million adults were hypertensive in 2005 and it is predicted to increase by about 60% to 1.56 billion in 2025 [2].

Existing evidence suggests that BP heritability ranges between 30-60% [3]. Despite the identification of specific causal genes involved in the regulatory pathways of rare familial forms of hypertension (HT) [4], both BP and HT are still considered polygenic traits involving a large number of metabolic pathways [5].

The complex architecture of BP regulation may account for the numerous discrepancies found among studies of the genetic background of HT. Inflammation, blood coagulation cascade, cellular adhesion molecules and lipid metabolism all appear to have significant roles [6-8]. While aspects of BP regulation may be the product of an inflammation stimulus [6,9], thrombin is also known to induce several intracellular pathways [7], to modulate vascular tone [7,10] and to have pro-inflammatory effects [7]. Growing evidence also suggests that cellular adhesion molecules and apolipoproteins are closely related to HT [8,11]. All of these factors may be involved in initiating and maintaining elevated BP levels [7]. Overall, however, the genetic factors associated with BP are poorly characterized and the effects of metabolic pathways highlighted by previous studies remain unclear.

The advent of high-throughput genotyping assays raised big expectations for a better identification of susceptibility BP loci [12-15] but limited progress has been made so far: the largest genome-wide association study (GWAS) published until now included ≈200,000 individuals and reported 28 loci associated with systolic BP (SBP), diastolic BP (DBP) and/or HT [12]. However, their risk score explained only 0.9% of BP phenotypic variance [12]. The persistence of this so-called ‘dark matter’ of BP genetic risk could be explained by the significant genetic and phenotypic heterogeneity of populations studied as well as the modest effect size of risk alleles [16]. In addition, conventional GWAS are based on independent gene/single nucleotide polymorphism (SNP) effects on a single phenotype without taking into account the possibility of two different genes/SNPs interactions that may affect the same trait. Studying regulatory mechanisms exerted by a given genetic variant modulating the effect of another i.e. epistatic interactions may be essential [17] in identifying the genes involved in BP regulation. Based on the above rationale [14,15,17], we hypothesize that research of epistatic interactions among candidate SNPs may represent a challenge in the search for disease-risk variants as the effect of one SNP may be altered or masked by the effects of another SNP. Furthermore, power to detect the first SNP is likely to be reduced and elucidation of the joint effects of two loci will be hindered by their interaction, unless explicitly examined. In fact, it is well-known that these interactions may identify genetic markers that are not captured by individual marker analysis and/or revealed by the combinatory effect of loci in other pathways [13,18].

As BP is a polygenic trait, where different metabolic pathways are involved (inflammation, coagulation cascade, sodium reabsorption, cellular adhesion and lipid metabolism), we regrouped 100 previously published cardiovascular candidate SNPs in 65 genes to perform a well-powered two-phases statistical analysis in two large independent populations in order to assess eventual epistatic interactions having an effect on BP.

## Methods

### Ethics statement

The samples are part of a human sample storage platform: the Biological Resources Bank (BRC) “Interactions Gène-Environnement en Physiopathologie CardioVasculaire” (IGE-PCV) in Nancy, East of France. All subjects gave written informed consent and the project protocol was approved by the local ethics committee of each centre.

### Study population

Both Discovery (phase 1) and Replication (phase 2) populations were homogenous and selected in the BRC IGE-PCV on the basis of the following criteria: (1) no antihypertensive drug therapy at recruitment; (2) complete clinical and genotypic data available; and (3) European origin.

The phase 1 enrolled 1,912 unrelated adults (51.13 ± 10.02 years, 51.3% women) recruited during free medical check-ups at the Center of Preventive Medicine of Vandœuvre-lès-Nancy in the East of France. They were Caucasians and born in France for three generations. As our purpose was to assess BP as a continuous trait, in a case-only design, normotensive and hypertensive subjects were included (for hypertensive individuals, data were gathered before the prescription of any medication).

The phase 2 enrolled 1,755 unrelated healthy Europeans from Crete (Greece); Belfast (Northern Ireland) and Lisbon (Portugal) (42.06 ± 9.89 years, 33% women) involved in the ApoEurope Project [19].

Clinical and biological data used for this study were collected at entrance before any eventual drug prescription following consultation for both populations.

### Clinical and biological data collection

SBP and DBP were measured under constant temperature (19°C-21°C) and standardized conditions (supine position) using a manual sphygmomanometer (*Colonne à mercure,* Mercurius) by expert nurses. The recorded values were the means of 3 readings on 20 min intervals. An adjustable BP cuff was used to correct errors due to variations in arm circumference [20]. All individuals underwent complete medical examination including anthropometric and biochemical measurements collected with standardized methods [21].

### Genotyping assays

Concerning the selection of assessed SNPs, 1/3 (35 SNPs in 15 genes) were chosen from the “Cardio-Vascular Disease 35” assay, a multi-locus genotyping assay developed in collaboration with Roche Molecular Systems [22]. These SNPs were candidate markers for CVD risk [22] specifically involved in the development and progression of atherosclerotic plaque. The 2/3 remaining were chosen based on the bibliography and on internal investigations performed predominantly in small to medium sized samples [7,23] (Additional file 1: Table S1).

Genotyping was performed using a multilocus assay with an immobilized probe approach designed by Roche Molecular Systems, Pleasanton, California, USA [22,24] and described in detail in the Additional file 1.

### PolyPhen analysis of nonsynonymous SNPs

The prediction of possible impact of nonsynonymous (ns) SNPs on the structure and function of their specific protein was performed using PolyPhen [25].

### Statistical analyses

Statistical analyses were performed using the SPSS® statistical software version 16.0 (SPSS, Inc, Chicago, Illinois) and Plink v01.7 [26].

Polymorphisms with minor allele frequencies (MAF) less than 2% or deviating from Hardy-Weinberg equilibrium (HWE) were excluded from individual analyses. However, for epistatic interaction analyses we included all SNPs as low MAF may identify an epistatic phenomenon.

The independent effect of the 100 genetic variants on SBP and DBP were determined assuming additive models using the minor allele as the reference group. Age, gender and body mass index (BMI)-adjusted linear regressions were performed and associations were considered significant when p-values adjusted for multiple testing (Bonferroni correction) were ≤ 0.05 and only if *a posteriori*-calculated statistical powers [calculated with SPSS® 16.0] were ≥ 80%.

We determined if the variants individually associated in our study were previously reported or in linkage-disequilibrium (LD) with variants previously reported in the literature, notably on GWAS. SNPs with a correlation coefficient ≥ 0.8 were considered in LD and then replicated.

Two-locus additive epistasis was defined as significant statistical interaction between two SNPs [27] with an *a posteriori*-calculated statistical power [calculated with SPSS® 16.0] ≥ 80%. All possible 2×2 combinations between the 100 SNPs involved in the discovery study (phase 1) were tested on a linear additive model (p-value ≤ 0.05) adjusted for age, gender and BMI in the Discovery population. In phase 2, similar models were applied for the replication of the two significant epistatic interactions observed in phase 1.

Values of SBP and DBP were log-transformed in order to normalise their distribution.

## Results

Characteristics of the study populations are presented in Table 1.

**Table 1:** Mean characteristics of studied individuals

**Characteristics**	**Discovery population**	**Replication population**
N (% women)	1,912 (51.3%)	1,755 (33%)
Age (years)	51.13 ± 10.02	42.06 ± 9.89
BMI (kg/m^2^)	26.01 ± 3.75	26.23 ± 4.11
SBP (mmHg)	142.86 ± 21.47	128.62 ± 17.69
DBP (mmHg)	87.49 ± 14.26	81.15 ± 10.93

BMI: body mass index, SBP: systolic blood pressure, DBP: diastolic blood pressure.

### Assessed genetic variants

All genes following MAF quality control were consistent with HWE (data not shown). Among the 100 SNPs investigated, 50 were nonsynonymous, and according to Polyphen [25], rs1800888, rs5742912, rs7412, rs5361 and rs2228570 were related to a modification in their corresponding protein structures (Additional file 1: Table S1).

### Individual association analysis

Associations among genetic variants and BP traits are shown in Table 2. Nine SNPs in 8 genes including different metabolic pathways were significantly associated with SBP or DBP: *LPL* and *LDLR* (lipid metabolism), *F7* (coagulation factor), *AGT1R* (BP regulation), *CSF2*, *IL1B*, *TGFB1* and *VDR* (inflammatory pathways). Seven SNPs were associated with both SBP and DBP levels; rs5186 in *AGT1R* (P_adjusted_≤0.027), rs25882 in *CSF2* (P_adjusted_≤3.83×10^-04^), rs5742910 in *F7* (P_adjusted_≤0.03), rs1143634 in *IL1B* (P_adjusted_≤0.049), rs5742911 in *LDLR* (P_adjusted_≤0.035) rs1800469 in *TGFB1* (P_adjusted_≤0.036) and rs2228570 in *VDR* (P_adjusted_≤9.48×10^-04^).

**Table 2:** Genetic variants associated with blood pressure

**Gene**	**OMIM accession number**	**SNP**	**Ancestral alleles**	**Type**	**MAF**	**p-value**	**p-value adjusted**	**Beta**	**SE**	**Trait**
*AGTR 1*	*106165	rs5186	A	3’UTR	28.933	8.34×10^-04^	0.027	−0.051	0.032	SBP
						6.96×10^-03^	0.041	−0.045	0.051	DBP
*CSF2*	*138960	rs25882	T	Exonic	25.596	4.67×10^-06^	3.83×10^-04^	0.124	0.030	SBP
						7.25×10^-07^	5.94×10^-05^	0.148	0.048	DBP
*F7*	227500	rs5742910	NA	Upstream	15.024	3.72×10^-04^	0.030	−0.028	0.041	SBP
						3.34×10^-04^	0.027	−0.042	0.065	DBP
		rs6046	C	Exonic	12.874	5.33×10^-04^	0.044	−0.028	0.044	SBP
*IL1B*	*147720	rs1143634	C	Exonic	19.397	4.21×10^-04^	0.035	−0.044	0.035	SBP
						5.92×10^-04^	0.049	−0.052	0.056	DBP
*LDLR*	606945	rs5742911	A	3’UTR	32.308	4.31×10^-04^	0.035	0.06	0.029	SBP
						1.17×10^-04^	0.010	0.071	0.047	DBP
*LPL*	609708	rs1800590	G	5’UTR	2.586	3.46×10^-04^	0.028	0.076	0.088	SBP
*TGFB1*	*190180	rs1800469	C	Upstream	29.048	4.44×10^-04^	0.036	−0.026	0.030	SBP
						9.77×10^-06^	8.02×10^-04^	−0.038	0.047	DBP
*VDR*	*601769	rs2228570	T	Exonic	40.139	1.16×10^-05^	9.48×10^-04^	−0.012	0.028	SBP
						6.02×10^-06^	4.93×10^-04^	−0.009	0.045	DBP

SNP: single nucleotide polymorphism, MAF: minor allele frequency, beta: regression coefficient corresponding to the minor allele of each SNP, SE: standard error, SBP: systolic blood pressure, DBP: diastolic blood pressure.

Age, gender and BMI were not significantly interacting with these SNPs.

Our imputation analyses showed that none of these significant SNPs have been reported or were in LD (r^2^ < 0.80) with genetic variants highlighted in previous GWAS [14,15].

### Epistatic interaction with BP

Epistatic interactions with BP are shown in Table 3.

**Table 3:** Epistatic interactions between genetic variants associated with blood pressure

A: Epistatic interactions in Discovery population betweenVCAM1 (rs1041163) * APOB (rs1367117) on SBP (P=0.04)					
SBP		***rs1041163***			
		11	12	22	N TOT
***rs1367117***	11	141.47 ± 21.31 (487)	138.55 ± 22.08 (213)	152.55 ± 19.69 (30)	730
	12	137.27 ± 21.22 (468)	140.67 ± 21.27 (182)	146.15 ± 20.68 (28)	678
	22	139.78 ± 19.24 (111)	135.43 ± 24.96 (41)	139.83 ± 26.73 (8)	160
	N TOT	1066	436	66	1568
B: Epistatic interactions in Discovery population betweenVCAM1 (rs1041163) * APOB (rs1367117) on DBP (P=0.027)					
DBP		***rs1041163***			
		11	12	22	N TOT
***rs1367117***	11	86.77 ± 14.47 (495)	85.35 ± 13.10 (232)	87.95 ± 14.22 (37)	764
	12	87.97 ± 14.05 (436)	92.11 ± 11.99 (149)	88.04 ± 13.41 (21)	606
	22	90.06 ± 14.08 (135)	87.92 ± 11.19 (55)	96.00 (8)	198
	N TOT	1066	436	66	1568
C: Epistatic interactions in Discovery population betweenSCGB1A1 (rs3741240) * LPL (rs1800590) on SBP (P=0.02)					
SBP		***rs3741240***			
		11	12	22	N TOT
***rs1800590***	11	87.66 ± 14.31 (846)	87.14 ± 14.25 (739)	87.95 ± 14.22 (217)	1802
	12	86.85 ± 13.89 (46)	94.14 ± 11.41 (34)	88.04 ± 13.41 (11)	91
	22	(0)	99.30 ± 2.45 (4)	96.00 (1)	5
	N TOT	892	777	229	1898
D: Epistatic interactions in Discovery population betweenSCGB1A1 (rs3741240) * LPL (rs1801177) on DBP (P=0.03)					
DBP		***rs3741240***			
		11	12	22	N TOT
***rs1801177***	11	87.66 ± 14.36 (856)	87.32 ± 14.22 (751)	88.05 ± 14.19 (219)	1826
	12	86.55 ± 12.38 (36)	92.91 ± 12.23 (26)	85.65 ± 13.72 (9)	71
	22	(0)	(0)	96.00 (1)	1
	N TOT	892	777	229	1898
E: Epistatic interactions in Replication population betweenVCAM1 (rs1041163) * APOB (rs1367117) on DBP (P=0.045)					
DBP		***rs1041163***			
		11	12	22	N TOT
***rs1367117***	11	81.73 ± 0.47 (509)	80.80 ± 0.74 (241)	85.00 ± 2.07 (29)	779
	12	80.88 ± 0.49 (441)	80.55 ± 0.84 (183)	78.25 ± 2.69 (30)	654
	22	80.05 ± 1.11 (92)	83.71 ± 1.81 (46)	83.16 ± 3.19 (6)	144
	N TOT	1042	470	65	1577
F: Epistatic interactions in Replication population betweenSCGB1A1 (rs3741240) * LPL (rs1801177) on DBP (P=0.05)					
DBP		***rs3741240***			
		11	12	22	N TOT
***rs1800590***	11	80.58 ± 0.41 (681)	81.62 ± 0.41 (684)	81.09 ± 0.90 (168)	1533
	12	83.60 ± 2.04 (29)	81.76 ± 1.94 (21)	86.31 ± 3.02 (11)	61
	22	60.00 (1)	78.75 ± 1.2 (2)	(0)	3
	N TOT	711	707	179	1597

DBP: diastolic blood pressure.

SBP: systolic blood pressure.

Mean value ± SE (sample size).

Two epistases: *VCAM1* (rs1041163, Upstream, chromosome 1) * *APOB* (rs1367117, exonic, chromosome 2) and *SCGB1A1* (rs3741240, 5’ UTR, chromosome 11) * *LPL* (rs1800590, 5’ UTR, chromosome 8) were observed for both SBP and DBP in the discovery set (P≤0.04 and P≤0.03 respectively). We intended to replicate these interactions in phase 2 and observed significant interactions for DBP only (P=0.045 and P=0.05 respectively). Age, gender and BMI had no significant effect on these interactions.

Although we found no clear interaction pattern between phases 1 and 2, these interactions were putatively functional according to PolyPhen.

## Discussion

Even at early stages, HT is a major cause of disability and death for millions of people worldwide [1]. Observational studies involving more than 1 million adults from 61 prospective studies have indicated that death from HT-associated diseases (both IHD and stroke) increases linearly from BP levels as low as 115/75 mmHg among middle age and elderly individuals [28]. Therefore, isolating genetic variations that influence BP may have major implications for public health; however, individual genes have only shown a modest effect so far [29]. It is therefore essential to study possible epistatic gene interactions in order to identify gene combinations that synergistically influence BP regulation and HT.

In the current study, we linked several SNPs belonging to diverse biological pathways (lipid metabolism, coagulation, BP regulation and inflammatory pathways) to BP variations. Genetic variation may be responsible for hypertensive phenotypes by altering either the structure of encoded proteins or gene expression [14]. Through an *in silico* analysis (PolyPhen), 5 SNPs were found to have possible functional effects through altering the corresponding protein structure (rs1800888, rs5742912, rs7412, rs5361 and rs2228570). In contrast, rs1800469 in *TGFB1* has been reported to be associated with increased *TGFB1* gene transcription and plasma protein levels [30], suggesting altered gene expression [30,31]. Similarly, rs5742910 and nsSNP rs6046 in *F7* have been previously reported to be associated with a 1/3 decrease in F7 plasma levels [7]. These 2 SNPs have also been previously associated with BP in a population derived from the BRC IGE-PCV [7]. Combined with previous data, our finding suggests that these 2 SNPs are associated with BP possibly due to modifications in gene expression and alterations in plasma protein levels.

Polymorphisms may also affect gene expression by disrupting microRNA binding (miRNA). For example, in the present study, rs5186 C allele of *AGTR1* disrupts binding of miR155 to its complementary *AGTR1* mRNA [32,33] resulting in an overall increase in gene expression. A molecular mechanism for miR155 interaction with its polymorphic target rs5186A (3’ UTR of *AGTR1*) has also been proposed [33].

Additionally, it is reported that rs1800590 polymorphism of *LPL* affects lipoprotein lipase enzyme function [34,35]. Similarly, the coding nsSNP rs2228570 in *VDR* introduces an amino acid substitution (Met1Thr) that may modify the structure of the encoded protein, according to Polyphen [25]. Thus, the influences of *LPL* and *VDR* polymorphisms on BP may be the result of modifications in their protein structure and resulting changes in protein activity.

Epistatic interactions may also play a pivotal role in the discrepancies found among genetic studies. There are numerous reports that these interactions may identify genetic markers that are not captured by individual marker analysis and/or revealed by the combinatory effect of loci in other pathways [13,18]. Herein we observed two epistatic interactions common for all BP traits: *VCAM1* (rs1041163) * *APOB* (rs1367117) and *SCGB1A1* (rs3741240) * *LPL* (rs1800590) (Table 3). Interestingly, only rs1800590 common allele was independently associated to an increase of SBP (P_adjusted_=0.028, SE=0.088, β=0.076; Table 2). Its combination with rs3741240 minor allele showed an inversion of this effect on SBP as common homozygous individuals had lower SBP than heterozygous. SNP rs3741240 also revealed that rs1800590 was associated to DBP as well.

This significant interaction between *SCGB1A1* (rs3741240) and *LPL* (rs1800590) and the subsequent inversion phenomenon were also observed in an independent population (phase 2) on DBP.

*In silico* review showed that rs3741240 is a potentially functional SNP inhibiting phospholipase A2 [36] and decreasing plasma levels of high-density lipoprotein cholesterol (HDL-C) [37]. Taken together, *LPL* and *SCGB1A1* may interact through HDL-C to influence BP.

The epistatic interactions that we may have identified between *VCAM1* (rs1041163) * *APOB* (rs1367117) highlighted original associations with BP as neither rs1041163 nor rs1367117 was independently associated to any of the traits studied. This highlighted the fact that important genetic regulation pathways could be missed when using conventional single SNP-trait approaches. Whereas rs1041163 is a nonfunctional SNP, rs1367117 may result in an altered protein structure according to PolyPhen. Human studies have reported significant correlations between Apolipoprotein B (APOB), the main component of low-density lipoprotein cholesterol, and VCAM1 in the context of HT. Hubel et al. [38] have reported significant correlations between soluble levels of APOB and VCAM1 in women with endothelial dysfunction and HT as a result of preeclampsia.

Nevertheless, previous candidate gene studies have revealed other epistatic interactions in BP phenotypes [18,39] in non-European populations. In a cross-sectional study conducted in 402 middle aged and elderly Japanese, Misono et al. [18] demonstrated that individuals carrying a combination of *ADRB2* Gly/Gly and *NOS3* Glu/Glu genotypes had higher hypertensive risk. Moreover, Kohara et al. [39] in a case–control study conducted in 9,700 subjects reported that epistatic interactions are associated with increased risk of HT. Combined with our observations, there is insights that numerous epistatic interactions involving multiple physiological pathways influence BP levels and HT risk.

### Strengths and limitations

Genetic interactions have been little investigated by the scientific community so far. Indeed, gathering large independent populations allowing to investigate these phenomenon is complicated as the MAF may differ.

The present study showed significant interactions in both phases 1 and 2 for only one of the traits: DBP, with no clear interaction pattern certainly due to the limited size of our replication population. However, our results are strengthened by the *in* silico approach (PolyPhen) and previous biochemical and molecular studies that have provided biological evidence for our putative functional epistatic interactions. Nevertheless, to confirm our epistatic interactions reproducibility, reported SNPs should be tested in a GWAS with high resolution arrays and higher genome-wide covering in a large population.

## Conclusions and perspectives

In summary, we identified (a) susceptibility genes associated with BP including *LPL* and *LDLR* (lipid metabolism), *F7* (coagulation factor), *AGT1R* (BP regulation), *TNF*, *IL1B*, *CSF2*, *TGFB1, LTA* and *VDR* (inflammation); (b) epistatic interactions between putative functional SNPs involved in lipid metabolism: *LPL* (rs1800590) and *APOB* (rs1367117), inflammation: *SCGB1A1* (rs3741240) and cellular adhesion: *VCAM1* (rs1041163). Our results were not previously reported in the literature (previous GWAS studies) and they were not in LD with other SNPs identified in these studies concerning BP. It is important to emphasize that these findings may provide insight into novel pathophysiological mechanisms underlying HT and they may provide future therapeutic targets.

## Abbreviations

APOB: Apolipoprotein B; BMI: Body mass index; BP: Blood pressure; BRC: Biological resources bank; CVD: Cardiovascular disease; DBP: Diastolic blood pressure; GWAS: Genome-wide association study; HDL-C: High-density lipoprotein cholesterol; HT: Hypertension; HWE: Hardy-Weinberg equilibrium; IGE-PCV: Interactions Gène-Environnement en Physiopathologie CardioVasculaire; IHD: Ischemic heart disease; LD: Linkage disequilibrium; MAF: Minor allele frequency; miRNA: microRNA; SBP: Systolic blood pressure; SNP: Single nucleotide polymorphisms.

## Competing interests

The authors declare no competing interest.

## Authors’ contributions

Conception and design: MGS, MYT, NCN, SES, SVS. Acquisition of data: GS, SVS. Analysis of data: MGS, NCN, SES. Interpretation of data: MGS, NCN, SES, SVS. Drafting of the manuscript: MGS, NCN, SES, SVS. Revision of the manuscript for important intellectual content: GS, MYT. All authors read and approved the final manuscript.

## Pre-publication history

The pre-publication history for this paper can be accessed here:

http://www.biomedcentral.com/1471-2350/14/2/prepub

## Supplementary Material

Additional file 1 **Genotyping assays. Table S1.** Summary of investigated genetic variants. **Table S2.** Minor allele frequency of the investigated SNPs in ApoEurope. Click here for file
