# Supplementary material for: Epistatic study reveals two genetic interactions in blood pressure regulation
Source: BMC Med Genet. 2013 Jan 8;14:2. doi: 10.1186/1471-2350-14-2 (PMC3599121; doi:10.1186/1471-2350-14-2)
Supplement: Additional file 1 — Genotyping assays. Table S1. Summary of investigated genetic variants. Table S2. Minor allele frequency of the investigated SNPs in ApoEurope. [file 1471-2350-14-2-S1.doc]

**Epistatic study reveals two genetic interactions in Blood Pressure regulation**

Additional file 1

**GENOTYPING ASSAYS**

Genotyping was performed using a multilocus assay with an immobilized probe approach designed by Roche Molecular Systems, Pleasanton, California, USA .

After PCR amplification using pooled biotinylated primers and hybridation to sequence-specific oligonucleotide probes, genotype assignments were performed by two independent observers using proprietary Roche Molecular Systems image processing software. Discordant results (< 3% of all scoring) were resolved by a third observer and if necessary, by a joint reading.

In order to replicate the significant epistatic interactions observed in the discovery set, SNPs of interest were genotyped in Kbioscience (http://www.kbioscience.co.uk) using the competitive allele specific PCR (KASP) chemistry coupled with a FRET-based genotyping system (http://www.kbioscience.co.uk/reagents /KASP/KASP.html) in the replication population (Additional file 1: Table S2).

Additional file 1: **Table S1: Summary of investigated genetic variants.**

| Locus | Gene  (OMIM accession number) | Chromosome | SNP ID | Position | Type | Mutation |
| --- | --- | --- | --- | --- | --- | --- |
| Blood pressure regulation | |  |  |  |  |  |
| ACE | Angiotensin I converting enzyme  (+106180) | 17q23.3 | rs1799752 | 17 :61565890-61565891 | Intronic | Ins/del |
| ADD1 | Adducin 1 (alpha)  (*102680) | 4p16.3 | rs4961** | 4 :2906707 | NsSNP-exonic | Gly460Trp |
| ADRB2 | Adrenergic, beta-2-, receptor, surface  (+109690) | 5q31-q32 | rs1042713* | 5 :148186633 | NsSNP-exonic | Arg16Gly |
|  |  |  | rs1042714* | 5 :148186666 | NsSNP-exonic | Gln27Glu |
|  |  |  | rs1800888*** | 5 :148187078 | NsSNP-exonic | Thr164Ile |
| AGT | Angiotensinogen (serpin peptidase inhibitor, clade A, member 8)  (+106150) | 1q42-q43 | rs699* | 1 :228912417 | NsSNP-exonic | Met235Thr |
| AGTR1 | Angiotensin II receptor, type 1  (*106165) | 3q21-q25 | rs5186 | 3 :148459988 | 3’UTR | 1166 A/C |
| GNB3 | Guanine nucleotide binding protein  (G protein), beta polypeptide 3  (*139130) | 12p13 | rs5443 | 12 :6954875 | Synonymous-exonic | Ser275Ser |
| NOS2 | Nitric oxide synthase 2, inducible  (*163730) | 17q11.2-q12 | rs1137933 | 17 :23130059 | Synonymous-exonic | Asp346Asp |
| NOS3 | Nitric oxide synthase 3 (endothelial cell)  (+163729) | 7q36 | rs1799983* | 7 :150696111 | NsSNP-exonic | Glu298Asp |
|  |  |  | rs1800779 | 7:150689943 | Intronic | -922 A/G |
| NPPA | Natriuretic peptide  (*108780) | 1p36.21 | rs5063 | 1:11907603 | Intronic | 664 G/A |
|  |  |  | rs5065 | 1:11906068 | Stop-exonic | 2238 T/C |
| SCNN1A | Sodium channel, nonvoltage-gated 1 alpha  (*600228) | 12p13 | rs5742912*** | 12 : 6328611 | Synonymous-exonic | Trp493Arg |
|  |  |  | rs2228576* | 12 : 6327323 | Synonymous-exonic | Thr663Ala |
| Lipid metabolism | |  |  |  |  |  |
| ADRB3 | Adrenergic, beta-3-, receptor  (*109691) | 8p12-p11.2 | rs4994* | 8 : 37942955 | Synonymous-exonic | Trp64Arg |
| APOA4 | Apolipoprotein A-IV4  (*107690) | 11q23 | rs675* | 11:116691675 | Synonymous-exonic | Thr347Ser |
|  |  |  | rs5110* | 11:116691634 | Synonymous-exonic | Gln360His |
| APOB | Apolipoprotein B (including Ag(x) antigen)  (+107730) | 2p24-p23 | rs1367117** | 2 : 21117405 | Synonymous-exonic | Thr71Ile |
|  |  |  | rs5742904** | 2 :  21082665 | Synonymous-exonic | Arg3500Gln |
| APOC3 | Apolipoprotein C-III  (+107720) | 11q23.1-q23.2 | rs2542052 | 11:116699984 | Upstream | -641 C/A |
|  |  |  | rs2854117 | 11:116700142 | Upstream | -482 C/T |
|  |  |  | rs2854116 | 11:116700169 | Upstream | -455 T/C |
|  |  |  | rs4520 | 11:116701535 | Synonymous-exonic | Gky34Gly |
|  |  |  | rs5128 | 11:116703640 | 3’UTR | 3175 C/G |
|  |  |  | rs4225 | 11:116703671 | 3’UTR | 3206 T/G |
| APOE | Apolipoprotein E  (+107741) | 19q13.2 | rs7412*** | 19:45412079 | NsSNP-exonic | Arg158Cys |
|  |  |  | rs429358* | 19:45411941 | NsSNP-exonic | Cys112Arg |
| CETP | Cholesteryl ester transfert protein, plasma  [+118470] | 16q21 | rs1800775 | 16:56995234 | Upstream | -629 C/A |
|  |  |  | rs5882* | 16:57016092 | NsSNP-exonic | Ile422Val |
|  |  |  | rs23037** |  | NsSNP-exonic | Asp442Gly |
|  |  |  | rs5742907 | 16:57016150 | Intron-essential splice site | 1 G/A |
| LDLR | Low density lipoprotein receptor  (+606945) | 19p13.3 | rs5742911 | 3’UTR | 19:11243445 | 1453A/G |
| LIPC | Lipase, hepatic  (+151670) | 15q21-q23 | rs1800588 | 15:58723675 | Intronic | -480 C/T |
| LPA | Lipoprotein, Lp(a)  (+152200) | 6q26 | rs7770628 |  |  | 93 C/T |
|  |  |  | rs1800769 | 6:161085267 | 5’UTR | 121 G/A |
| LPL | Lipoprotein lipase  (+609708) | 8p22 | rs1800590 | 8:19796671 | 5’UTR | -93 T/G |
|  |  |  | rs1801177* | 8:19805708 | NsSNP-exonic | Asp9Asn |
|  |  |  | rs268* | 8:19813563 | Stop-exonic | Asn291Ser |
|  |  |  | rs328 | 8:19819724 | NsSNP-exonic | Ser447Ter |
| PON1 | Paraoxonase 1  (+168820) | 7q21.3 | rs854560* | 7:94946067 | NsSNP-exonic | Met55Leu |
|  |  |  | rs662* | 7:94937439 | Stop-exonic | Gln192Arg |
| PON2 | Paraoxonase 2  (*602447) | 7q21.3 | rs7493* | 7:95034775 | NsSNP-exonic | Ser311Cys |
| PPARG | Peroxisome proliferator-  activated receptor gamma  (*601487) | 3p25 | rs1801282* | 3 : 12368125 | NsSNP-exonic | Pro12Ala |
| Cellular adhesion | |  |  |  |  |  |
| ICAM1 | Intercellular adhesion molecule 1  (*147840) | 19p13.3-p13.2 | rs1799969* | 19:10394792 | NsSNP-exonic | Gly241Arg |
|  |  |  | rs5491* | 19:10385540 | NsSNP-exonic | Lys56Met |
| SELE | Selectin E  (+131210) | 1q22-q25 | rs5361*** | 1:169701060 | NsSNP-exonic | Ser149Arg |
|  |  |  | rs5355** | 1:169695870 | NsSNP-exonic | Leu554Phe |
| SELP | Selectin P (granule membrane protein 140kDa, antigen CD62)  (+173610) | 1q22-q25 | rs6131* | 1 :167847509 | NsSNP-exonic | Ser330Asn |
|  |  |  | rs6133* | 1 : 167831970 | NsSNP-exonic | Val640Leu |
| VCAM1 | Vascular cell adhesion molecule 1  (*192225) | 1p32-p31 | rs1041163 | 1:101183824 | Upstream | -1594 T/C |
| Homocystein metabolism | |  |  |  |  |  |
| CBS | Cystathionine beta-synthase  (*613381) | 21q22.3 | rs5742905** | 21 : 43356253 | NsSNP-exonic | Ile278Thr |
| MTHFR | Methylentetrahydrofolate reductase (NAD(P)H)  (*607093) | 1p36.3 | rs1801133** | 1:11856378 | NsSNP-exonic | Ala222Val |
| Coagulation cascade |  |  |  |  |  |  |
| F2 | Coagulation factor II (thrombin)  (*176930) | 11p11 | rs1799963 | 11:46761055 | 3’UTR | 20210 G/A |
| F5 | Coagulation factor V (proaccelerin, labile factor)  (*612309) | 1q23 | rs6025** | 1 : 167785673 | NsSNP-exonic | Arg506Gln |
| F7 | Coagulation factor VII (serum prothrombin conversion accelerator)  (+227500) | 13q34 | rs5742910 | 13: 113759833- 113759834 | Upstream | -323 Del/ins |
|  |  |  | rs6046* | 13 : 112821160 | NsSNP-exonic | Arg353Glu |
| FGB | Fibrinogen beta chain  (*134830) | 4q28 | rs1800790 | 4:155483708 | Upstream | -455 G/A |
| ITGA2 | Integrin, alpha 2 (CD49B, alpha 2 subunit of VLA-2 receptor)  (+192974) | 5q23-q31 | rs1062535 | 5:52351413 | Synonymous-exonic | Thr275Thr |
| ITGB3 | Integrin, beta 3 (platelet glycoprotein IIIa, antigen CD61)  (+173470) | 17q21.32 | rs5918* | 17 : 42715729 | NsSNP-exonic | Leu33Pro |
| SERPINE1 | Serpin peptidase inhibitor,  calde E (Nexin plasminogen activator inhibitor type 1), member 1  (*173360) | 7q21.3-q22 | rs7242 | 7:100781445 | 3’UTR | 11053 G/T |
|  |  |  | rs1799768 | 7:100769707-10076970 | Upstream | (-675) 5G/4G |
| Inflammation | |  |  |  |  |  |
| C3 | Complement component 3  (+120700) | 19p13.3-p13.2 | rs2230199* | 19 : 6669387 | NsSNP-exonic | Arg102Gly |
| C5 | Complement component 5  (+120900) | 9q33-q34 | rs17611* | 19 : 122809021 | NsSNP-exonic | Ile802Val |
| CCL11 | Chemokine (C-C motif) ligand 11  (*601156) | 17q21.1-q21.2 | rs4795895 | 17:32611446 | Upstream | -1328 G/A |
|  |  |  | rs1129844 * | 17:32612894 | NsSNP-exonic | Ala23Thr |
| CCR2 | Chemokine (C-C motif) receptor 2  (*601267) | 3p21.31 | rs1799864* | 3:46399208 | NsSNP-exonic | Val62Ile |
| CCR3 | Chemokine (C-C motif) receptor 3  (*601268) |  | rs5742906** | 3:46306765 | NsSNP-exonic | Pro39Leu |
| CCR5 | Chemokine (C-C motif) receptor 5  (*601373) | 3p21.31 | rs333 | 3:46414947-46414978 | Frameshift coding-exonic | wt/Δ580-611 |
|  |  |  | rs1799987 | 3:46411935 | Intronic | -2454 G/A |
| CD14 | CD14 molecule  (*158120) | 5q31.1 | rs2569190 | 5:140012916 | 5’UTR | -260 C/T |
| CSF2 | Colony stimulating factor 2 (granulocyte-macrophage)  (*138960) | 5q31.1 | rs25882* | 5 : 131439359 | NsSNP-exonic | Ile117Thr |
| CTLA4 | Cytotoxic T-lymphocyte-associated protein 4  (+123890) | 2q33 | rs5742909 | 2:204732347 | Upstream | -318 C/A |
|  |  |  | rs231775* | 2:204732714 | NsSNP-exonic | Thr17Ala |
| CXCL12 | Chemokine, (C-X-C motif), ligand 12  (*600835) | 10q11.1 | rs1801157 | 10:44868257 | 3’UTR | 800 G/A |
| GC | Group-specific component  (vitamin D binding protein)  (+139200) | 4q12-q13 | rs7041* | 4: 72837198 | NsSNP-exonic | Glu416Asp |
| IL1A | Interleukin 1, alpha  (*147760) | 2q14 | rs1800587 | 2:113542960 | Upstream | -889 T/C |
| IL1B | Interleukin 1, beta  (*147720) | 2q14 | rs1143634 | 2 :113306861 | Synonymous-exonic | Phe105Phe |
|  |  |  | rs16944 | 2:113594867 | Upstream | -1418 C/T |
| IL4 | Interleukin 4  (*147780) | 5q31.1 | rs2243250 | 5:132009154 | Upstream | -590 C/T |
| IL4R | Interleukin 4 receptor  (*147781) | 16p12.1-p11.2 | rs1805010** | 16: 27355703 | Synonymous-exonic | Ile50Val |
|  |  |  | rs1805015** | 16: 27373680 | Synonymous-exonic | Ser478Pro |
|  |  |  | rs1801275** | 16:27374400 | Synonymous-exonic | Gln576Arg |
| IL5RA | Interleukin 5 receptor, alpha  (*147851) | 3p26-p24 | rs2290608 | 3:3151759 | 5’UTR | -80 G/A |
| IL6 | Interleukin 6 (interferon, beta 2)  (*147620) | 7p21 | rs1800795 | 7:22766645 | Intronic | -174 G/C |
|  |  |  | rs1800796 | 7:22766246 | Intronic | -572 G/C |
| IL9 | Interleukin 9  (*146931) | 5q31.1 | rs2069885* | 5 :135256064 | Synonymous-exonic | Thr113Met |
| IL10 | Interleukin 10  (*124092) | 1q31-q32 | rs1800872 | 1:206946407 | Upstream | -571 C/A |
| IL13 | Interleukin 13  (*147683) | 5q31 | rs1295686 | 5:131995843 | Intronic | 4045 C/T |
| LTA | Lymphotoxin alpha (TNF superfamily, member 1)  (+153440) | 6p21.3 | rs909253 | 6 :31540313 | 5’UTR | 252 A/G |
| LTC4S | Leukotriene C4 synthase  (+246530) | 5q35 | rs730012 | 5:179220638 | 3’UTR | -444 A/C |
| MMP3 | Matrix metallopeptidase 3 (stromelysin 1, progelatinase)  (+185250) | 11q22.3 | rs3025079 |  |  | (-1171) 5A/6A |
| TCF7 | Transcription factor 7 (T-cell specific, HMG-box)  (*189908) | 5q31.1 | rs5742913* | 5: 133451183 | Synonymous-exonic | Pro19Ter |
| TGFB1 | Transforming growth factor, beta 1  (*190180) | 19q13.1 | rs1800469 | 19:41860296 | Upstream | -509 C/T |
| TNF | Tumor necrosis factor  (*191160) | 6p21.3 | rs1800629 | 6 :31651010 | Upstream | -308 G/A |
|  |  |  | rs361525 | 6 :31651080 | Upstream | -238 G/A |
|  |  |  | rs1041981* | 6 :31648763 | Synonymous-exonic | Thr26Asn |
| SCGB1A1 | Secretoglobin, family 1A, member 1 (uteroglobin)  (*192020) | 11q12.3-q13.1 | rs3741240 | 11 :61943118 | 5’UTR | 38 G/A |
| VDR | Vitamin D  (1,25-dihydroxyvitamin D3) receptor  (*601769) | 12q13.11 | rs1544410 | 12:48239835 | INTRONIC | 63980G>A |
|  |  |  | rs2228570*** | 12 :48272895 | Synonymous-exonic | Met1Thr |

The prediction of possible nsSNPs impact on the structure and function of its specific protein was performed using PolyPhen.

*: benign nsSNP.

**: possibly damaging nsSNP.

***: damaging nsSNP.

nsSNP.: non synonymous single nucleotide polymorphism

Additional file 1: **Table S2: Minor allele frequency of the investigated SNPs in ApoEurope**

| **SNP** | **GENE** | **MAF** |
| --- | --- | --- |
| rs1041163 | *VCAM1* | 20.2 |
| rs1367117 | *APOB* | 19 |
| rs1800590 | *LPL* | 1.9 |
| rs3741240 | *SCGB1A1* | 31.8 |
